# Diverse and complex developmental mechanisms of early Ediacaran embryo-like fossils from the Weng'an Biota, southwest China

**DOI:** 10.1098/rstb.2021.0032

**Published:** 2022-03-28

**Authors:** Zongjun Yin, Weichen Sun, Pengju Liu, Junyuan Chen, David J. Bottjer, Jinhua Li, Maoyan Zhu

**Affiliations:** ^1^ State Key Laboratory of Palaeobiology and Stratigraphy, Nanjing Institute of Geology and Palaeontology, Chinese Academy of Sciences, Nanjing 210008, People's Republic of China; ^2^ Centre for Excellence in Life and Palaeoenvironment, Chinese Academy of Sciences, Nanjing 210008, People's Republic of China; ^3^ Nanjing College, University of Chinese Academy of Sciences, Nanjing 211135, People's Republic of China; ^4^ College of Earth and Planetary Sciences, University of Chinese Academy of Sciences, Beijing 100049, People's Republic of China; ^5^ University of Science and Technology of China, Hefei 230026, People's Republic of China; ^6^ Institute of Geology, Chinese Academy of Geological Sciences, Beijing 100037, People's Republic of China; ^7^ Department of Earth Sciences, University of Southern California, Los Angeles, CA 90089, USA; ^8^ Key Laboratory of Earth and Planetary Physics, Institute of Geology and Geophysics, Innovation Academy for Earth Science, Chinese Academy of Sciences, Beijing 100029, People's Republic of China

**Keywords:** Ediacaran, embryo-like fossils, Weng'an Biota, holozoan, cell division patterns

## Abstract

The origin and early evolution of animal development remain among the many deep, unresolved problems in evolutionary biology. As a compelling case for the existence of pre-Cambrian animals, the Ediacaran embryo-like fossils (EELFs) from the Weng'an Biota (approx. 609 Myr old, Doushantuo Formation, South China) have great potential to cast light on the origin and early evolution of animal development. However, their biological implications can be fully realized only when their phylogenetic positions are correctly established, and unfortunately, this is the key problem under debate. As a significant feature of developmental biology, the cell division pattern (CDP) characterized by the dynamic spatial arrangement of cells and associated developmental mechanisms is critical to reassess these hypotheses and evaluate the diversity of the EELFs; however, their phylogenetic implications have not been fully realized. Additionally, the scarcity of fossil specimens representing late developmental stages with cell differentiation accounts for much of this debate too. Here, we reconstructed a large number of EELFs using submicron resolution X-ray tomographic microscopy and focused on the CDPs and associated developmental mechanisms as well as features of cell differentiation. Four types of CDPs and specimens with cell differentiation were identified. Contrary to the prevailing view, our results together with recent studies suggest that the diversity and complexity of developmental mechanisms documented by the EELFs are much higher than is often claimed. The diverse CDPs and associated development features including palintomic cleavage, maternal nutrition, asymmetric cell divisions, symmetry breaking, establishment of polarity or axis, spatial cell migration and differentiation constrain some, if not all, EELFs as total-group metazoans.

This article is part of the theme issue ‘The impact of Chinese palaeontology on evolutionary research’.

## 1. Introduction

Molecular clock estimates indicate that animals probably originated before the Cryogenian [1,2], but the current fossil record does not support this proposal well [3]. The exceptionally preserved Ediacaran embryo-like fossils (EELFs) with cellular and subcellular structures have great potential to deepen our understanding of the gap between the fossil records and the molecular clock estimates, since they were initially described as animal embryos [4] and thought to ‘open a new era in the study of early animal evolution’ [5, p. 529]. This interpretation has been supported by many later studies [6–16]. And what is more important, the age of the EELFs, roughly around 610 Myr old [17], is much earlier than the celebrated, megascopic Ediacaran Biota.

**Figure 1.**
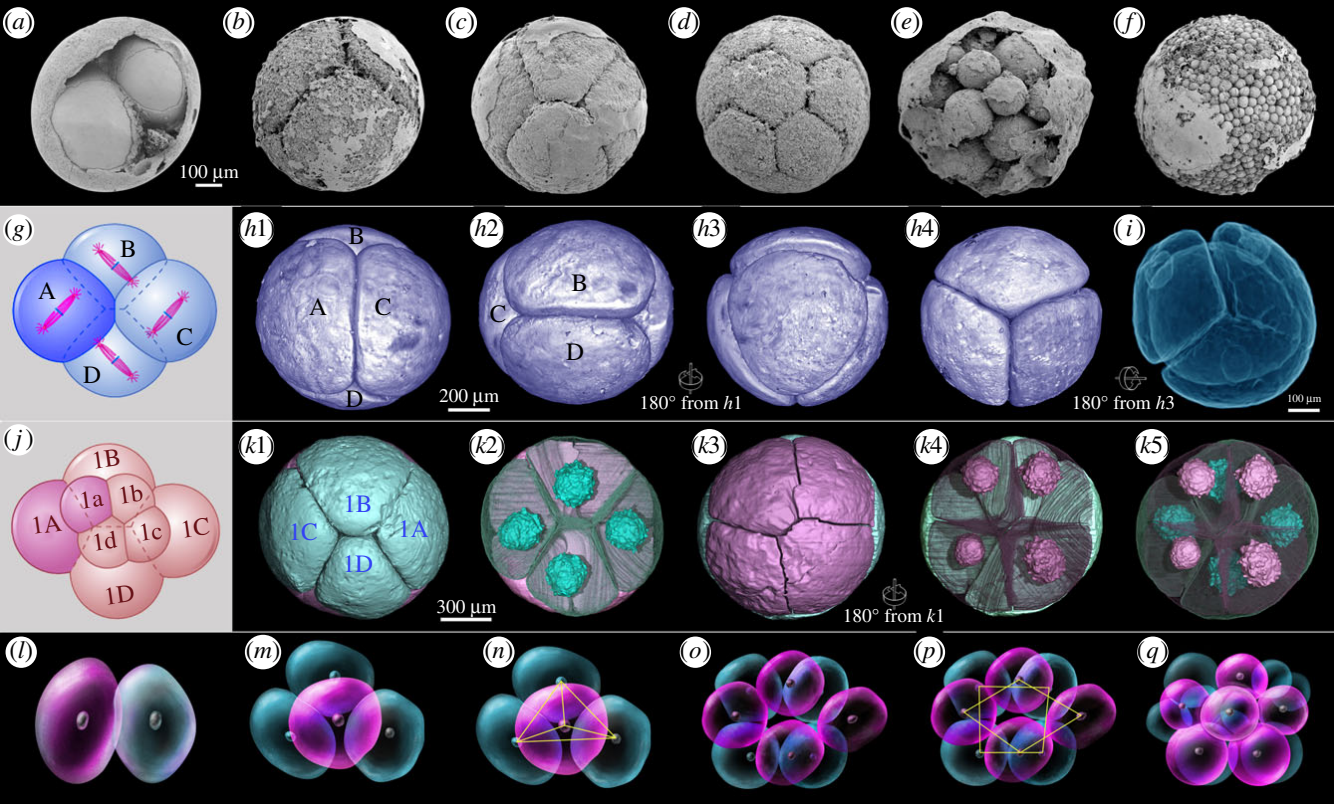
Ediacaran embryo-like fossils with equal and synchronous cell division and their extant analogues. (*a*–*f*) Fossil embryos with equal and synchronous cleavage at different stages. (*g*,*j*) Diagrams showing 4- and 8-celled embryos with spiral cleavage pattern; (*h*1–*h*4) a 4-celled fossil embryo from different views. (*i*) Transparent model for a 4-celled fossil embryo. (*k*1–*k*5) An 8-celled fossil embryo from different views. Note the well-preserved nuclei displayed in (*k*2), (*k*4) and (*k*5). (*l*–*q*) Early cleaving process of living sponge *Spongilla lacustris* (after [19,20]).

The most abundant EELFs from the Weng'an Biota are spherical with diameter around 450–800 µm [18]. During cell division, their total volumes remain unchanged while the daughter cells increase in number and decrease in volume (figure 1). Cell division without cytoplasmic growth, i.e. palintomic cleavage, can be found in metazoan embryos. And largely because of this feature and the 2*^n^* pattern of cell number growth, the EELFs were interpreted as animal embryos [4,11]. However, the animal interpretation has been challenged by alternative hypotheses, including giant bacteria [21], non-metazoan holozoans (mesomycetozoan-like protists) [22], stem metazoans [23,24] or multicellular algae [24,25], because, at least in part, palintomic cleavage is not an exclusive characteristic of animal embryos, and it can also be found within some non-metazoan holozoans (e.g. mesomycetozoans, also known as ichthyosporeans) and green algae (e.g. volvocine algae) [25–27].

The debate on the affinities of the EELFs largely derives from a viewpoint that these cleaving EELFs were morphologically simple with extremely low diversity [22], yielding very few phylogenetic signals [18,22,26]. The EELFs with equal and synchronous cleavage [4] have long been thought to represent the whole story of the EELFs from the Weng'an Biota [22,26]. A few previous studies have reported EELFs with distinctive cell division patterns (CDPs), including polar-lobe formation [8,15] and meroblastic cleavage [16], but the significance of these discoveries has not been fully realized [18,26]. Additionally, animal adult forms reported previously, including small bilaterian *Vernanimalcula* [28] and tubular cnidarians [29], are not widely accepted [26,30–32], leading to a conclusion that these abundant EELFs are non-metazoan protists without complex later developmental stages [22,26]. This viewpoint was challenged by a discovery of some EELFs (*Megaclonophycus*-stage *Megasphaera*) with ‘matryoshkas’ which were interpreted as stem-group metazoans with cell differentiation and germ–soma separation [24]. But this hypothesis is likewise controversial [26,33] (but see [34]). More recent studies on EELFs such as *Helicoforamina* [35] and *Caveasphaera* [36] revealed cryptic diversity and holozoan affinity for these EELFs. However, it is still unclear how diverse the EELFs are. Furthermore, the exact phylogenetic positions within the holozoan tree for different EELFs remain contentious.

To test these competing hypotheses, we reconstructed a large number of the Weng'an EELFs using submicron resolution X-ray tomographic microscopy and scanning electron microscopy. Our new results not only are helpful to understanding the biodiversity of the EELFs, but also provide us significant evidence to reconstruct their developmental sequences and constrain their phylogenetic positions.

## 2. Results

### (a) Type 1: Ediacaran embryo-like fossils with equal and synchronous cleavage

The fossils with equal and synchronous cleavage (figure 1*a–f*) were the first EELFs reported from the Weng'an Biota [4,11]. They were assigned into a morphological taxon, *Megasphaera* [11,37]. As iconic members of the Weng'an Biota, however, the CDP of these EELFs was poorly understood [4,18,23]. In addition to palintomic cleavage, these EELFs have several distinctive features. First, they usually have a single-layered smooth (figure 1*a*–*c*,*e*,*f*) or sculptured envelope (electronic supplementary material, figure S1 K–Q) [38] (but see an exception in Fig. 22.8 of [37]) with an even number of equal-sized cells inside, suggesting that they were undergoing equal and synchronous cleavage. This is why the number of the cells is equal to 2*^n^* (*n* = 0, 1, 2, 3…), and the daughter cells are always equal to each other in size [4,23]. Second, the geometric relationships between the cells at early cleaving stages are regular, stable and consistent (figure 1, electronic supplementary material, figure S1). For example, the cells are always organized as a tetrahedron at the 4-cell stage (figure 1*h*,*i*), and the 4-, 8- and 16-celled specimens have specific spatial arrangements of cells similar to extant animal embryos with spiral cleavage (figure 1*g*–*k*). Third, a large number of specimens possess large intracellular structures (LISs) (figure 1*k*) and small spherical granules within the cells. The origin of the LISs has been much more contentious [7,22,23,39]; however, based on high-resolution reconstructions and computed tomographic quantitative analysis as well as comprehensive taphonomic analysis, they have been shown to be cell nuclei [40,41].

### (b) Type 2: EELFs with equal and asynchronous cleavage

**Figure 2.**
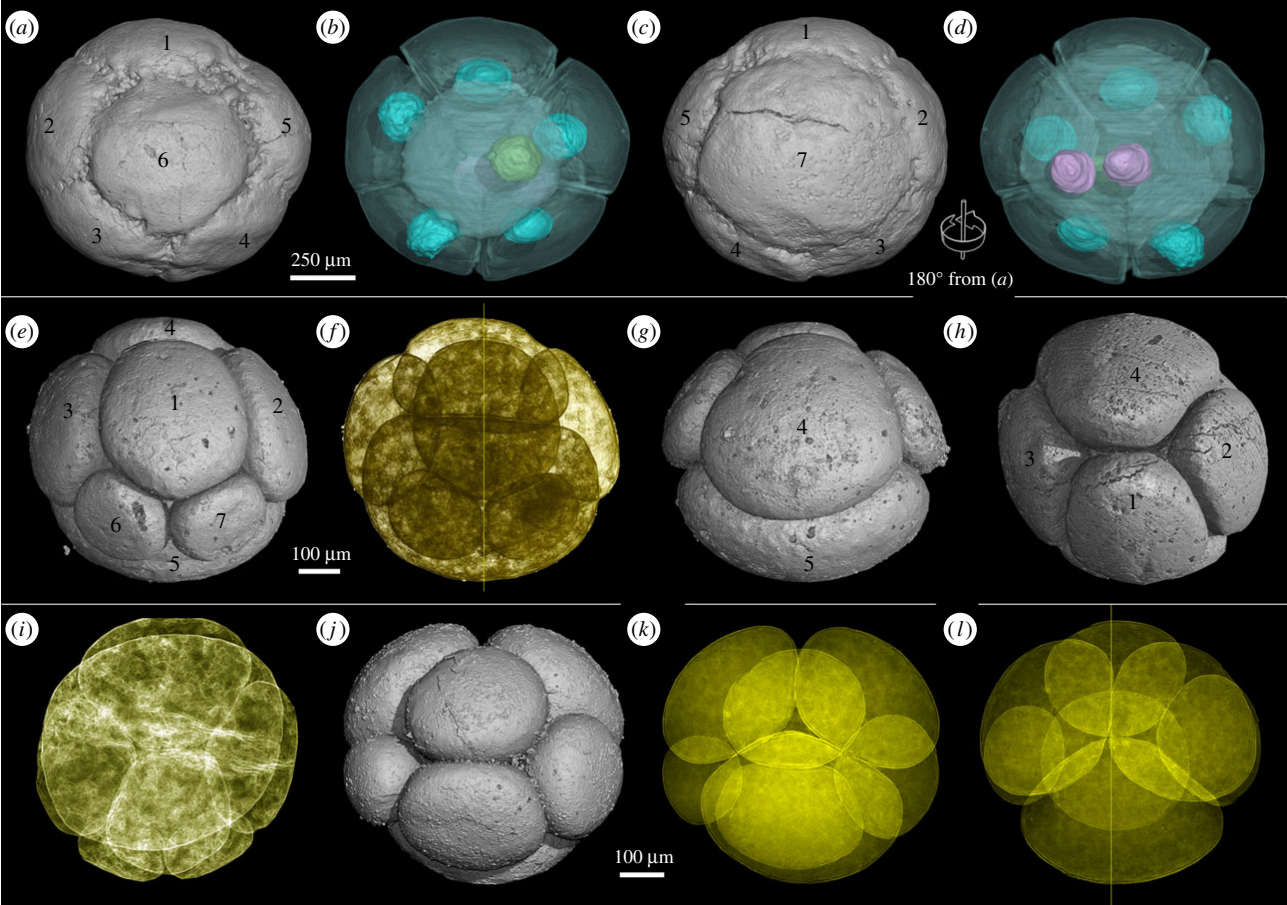
Ediacaran embryo-like fossils with asynchronous cleavage (*a*–*d*) and bilateral cell arrangement (*e*–*l*). (*a*–*d*) A 7-celled specimen with well-preserved nuclei. (*a*,*c*) Surface renderings with different views. (*b*,*d*) Transparent renderings of (*a*) and (*c*), respectively. Note that the small cells (cell-1 to cell-6) possess one nucleus each (green and cyan in (*b*)) while the large cell-7 possesses two nuclei (pink in (*d*)). (*e*–*i*) A specimen at 7-cell stage from different views. (*i*,*f*) Transparent renderings showing bilateral cell arrangement. (*j*–*l*) A 7-celled specimen. (*j*) Surface rendering, (*k*,*l*) transparent renderings from different angles.

Some EELFs with equal but asynchronous cleavage are also common in the Weng'an Biota [23]. At each division, the daughter cells are equal-sized, but one cell divided slower than the others, giving rise to a temporary large cell that is twice as big as the others. For example, the specimen illustrated in figure 2*a*–*d* has six small cells and a large cell [40]. Quantitative computed tomographic analysis shows that the large cell is almost twice as big as the other cells (electronic supplementary material, table S1). Furthermore, the large cell possesses two nuclei, and each nucleus has a similar size to the other nuclei in the six small cells (electronic supplementary material, table S1). All the data suggest that the large cell is ready for the third round of cytokinesis while the other six cells have finished the third round of cell division. In summary, the EELFs with equal and asynchronous cell division usually have an uneven number of cells, and the cell with a slower pace of division is twice as large as the others. Apart from asynchronous cell division, the other features, including spatial arrangement of cells, cell compaction and preservation of nuclei, are similar to those of type 1 EELFs.

### (c) Type 3: EELFs with bilaterally symmetric cell arrangement

Here are shown for the first time some EELFs from the Weng'an Biota with a bilaterally symmetric cell arrangement. For example, the two specimens in figure 2*e*–*l* were undergoing asynchronous cell division, and this is why the cell number of each of the two specimens is seven (cell number does not fit 2*^n^* pattern). Furthermore, the cleaving cells in the two specimens are not equal in size, and they can be assigned into three classes in terms of size. For instance, among the seven cells of the specimen illustrated in figure 2*e*, cell-5 is the largest one, and cells 1, 2, 3 and 4 are intermediate, while cells 6 and 7 are the smallest ones. When compared with the 7-celled specimen in figure 2*a*–*d*, it is apparent that the relative cell sizes and arrangement in the two 7-celled specimens displayed in figure 2*e*–*l* are different, suggesting that the unequal sizes of the cells result from both asynchronous and unequal cell divisions. What is more important, the spatial cell arrangement of the two specimens displays obvious bilateral symmetry, which is different from that of any EELFs reported previously. This bilaterally symmetric cell arrangement reflects potential developmental regulation on the orientations of cell division planes, suggesting the establishment of polarity or axis in this type of EELFs.

### (d) Type 4: EELFs with unequal and asynchronous cleavage

**Figure 3.**
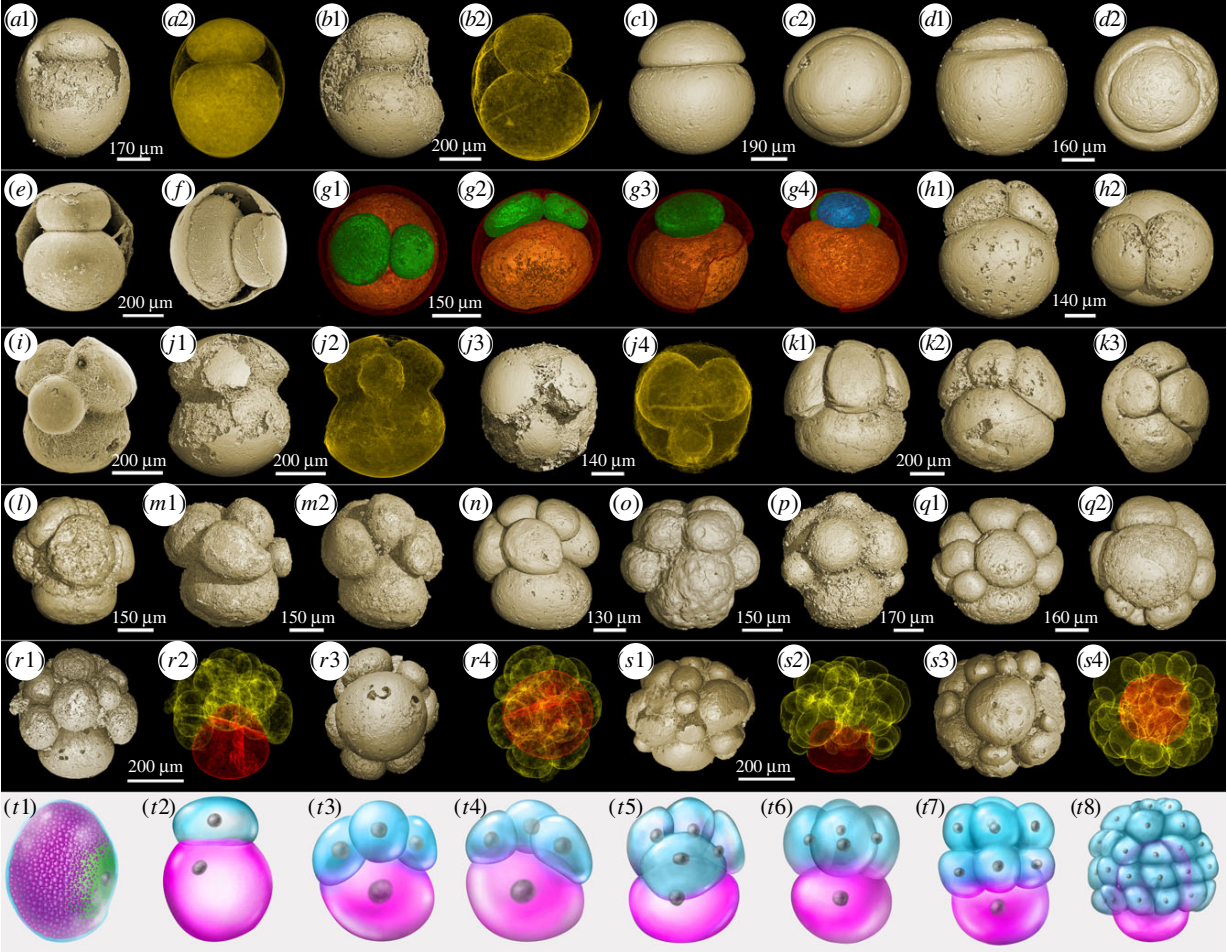
Ediacaran embryo-like fossils undergoing unequal and asynchronous cleavage and their extant analogue. (*a*–*f*) Different views of 2-cell stage specimens; (*g*,*h*) different views of 3-cell stage specimens; (*g*1–*g*4) transparent views showing internal structures; (*i*,*j*) different views of 4-cell stage specimens; (*k*1–*k*3) different views of a 5-cell stage specimen; (*l*) a 6-cell stage specimen; (*m*1,*m*2) different views of a 7-cell stage specimen; (*n*,*o*) 8-cell stage specimens; (*p*) a 9-cell stage specimen; (*q*1,*q*2) different views of a 10-cell stage specimen; (*r*1–*r*4) different views of a 14-cell stage specimen; (*s*1–*s*4) different views of a 25-cell stage specimen. (*a*2), (*b*2), (*j*2), (*r*2), (*r*4), (*s*2) and (*s*4) are transparent renderings of (*a*1), (*b*1), (*j*1), (*j*3), (*r*1), (*r*3), (*s*1) and (*s*3), respectively. The specimens in (*e*) and (*f*) are scanning electron microscopy images, and the others are tomographic data. (*t*1–*t*8) Early cleaving process of living embryos of rotifer *Asplanchna ebbesbornii* (after [42]). (*t*1) Fertilized zygote with abundant yolk granules (coloured red); (*t*2) 2-cell stage; (*t*3,*t*4) different views of 4-cell stage; (*t*5,*t*6) different views of 5-cell stage; (*t*7) 8-cell stage; (*t*8) late stereoblastula stage. The largest cell in each developmental stage is indicated in red.

Here, we report a unique group of EELFs with unequal, asynchronous cleavage, and show specific cell arrangement patterns (figure 3). Two specimens of this type of EELF were reported from the Weng'an Biota [6]; however, they have been overlooked because they lack detailed investigation. Based on a large number of specimens, we established a developmental sequence marked by unequally asynchronous cell division and a traceable large polar cell through different developmental stages (figure 3). Synchrotron and nanofocus tomographic microscopy reveal several distinct features of these fossils. First, similar to other EELFs, their cell division is by palintomic cleavage, but the number of cells does not follow the typical 2*^n^* pattern because of the asynchronous cell division. Instead, the cell number increases from 1 to 2, and then to 3, 4, 5 and so on. Second, the asymmetric cell division started to happen from the first round of cleavage and continued to much later stages, giving rise to a giant cell. The small cells formed a cap-like structure and covered the giant cell. Therefore, each specimen has a polarity marked by the axis through the cap of small cells to the giant cell. Third, the size of the giant cell decreased gradually from early to later cleaving stages (the diameter ratio of the giant cell to the whole specimen decreased from *ca* 89% at the 2-cell stage to 53% around the 15-cell stage), but it always was relatively larger than the rest of the cells at each cleaving stage, making it traceable through each developmental stage. Meanwhile, the cap of small cells grew larger from early to later stages and eventually embraced the giant cell, probably via cell migration (figure 3*r*,*s*). Fourth, most specimens illustrated in figure 3 are naked, without envelopes, but the complete specimens in fact possess bi-layered envelopes, and the inner layer is thin and smooth (figure 3*a*,*b*,*e*,*f*; electronic supplementary material, figure S2A–D) while the outer layer is thick and ornamented (electronic supplementary material, figure S2B–D), making them different from the type 1 EELFs (electronic supplementary material, figure S1), though both specimens have been assigned to the same morphological taxon, *Megasphaera ornata* [18]. Finally, exceptionally preserved subcellular granules with spherical or oval shapes can be observed in the majority of these EELFs (electronic supplementary material, figure S3). Different from fossilized nuclei, the granules always appear in large number, and have much smaller sizes, ranging from less than 10 µm to about 50 µm. The tomographic data suggest that the granules were coated by a membrane (electronic supplementary material, figure S3I–L). These coated granules have been widely interpreted as nutrient sources such as yolk granules or lipid droplets [23,40].

### (e) Type 5: elongate EELFs developing from cleaving embryos to late stages with cell differentiation

**Figure 4.**
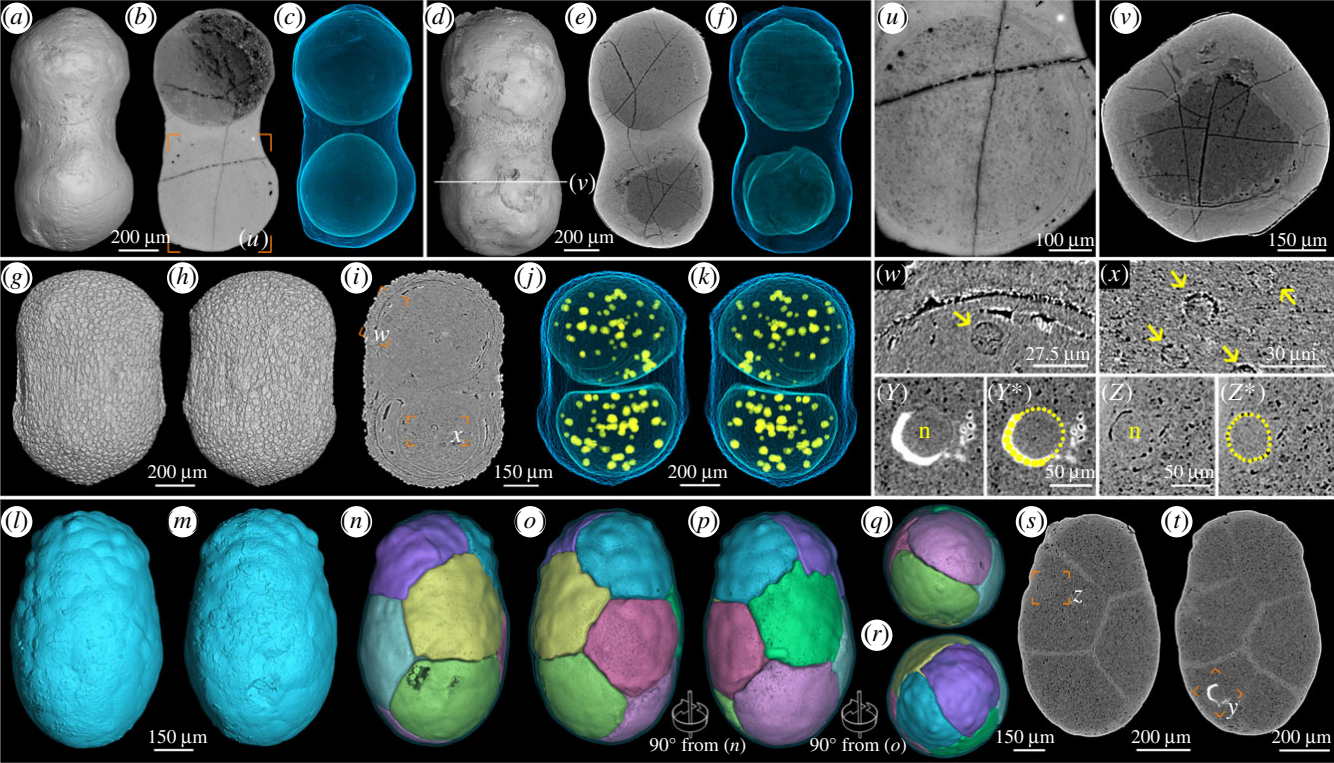
Elongated embryo-like fossils at early cleavage stages. (*a*–*k*) Three specimens at 2-cell stage. (*l*–*r*) A specimen at 8-cell stage. (*a*,*d*,*g*,*h*,*l*,*m*) Surface renderings. (*b*,*e*,*i*,*s*,*t*) Orthoslices showing cell division inside. (*c*,*f*,*j*,*k*) Transparent renderings showing internal cells; note the small spherical intercellular structures in (*j*) and (*k*). (*n*–*r*) Volume renderings from different perspectives, showing the cleavage pattern of the cells. (*u*,*w*–*z*) Close-up views of the framed areas in (*b*),(*i*),(*s*) and (*t*); (*v*) cross-section with position indicated in (*d*); (*u*,*v*) displaying the deformed cell boundaries altered during post-mortem and diagenetic process; (*w*,*x*) showing details of small intercellular granules (arrows); (*y*,*z*) showing cell nuclei—the boundaries of the nuclei are indicated by dotted lines in (y*) and (z*). n, nucleus.

**Figure 5.**
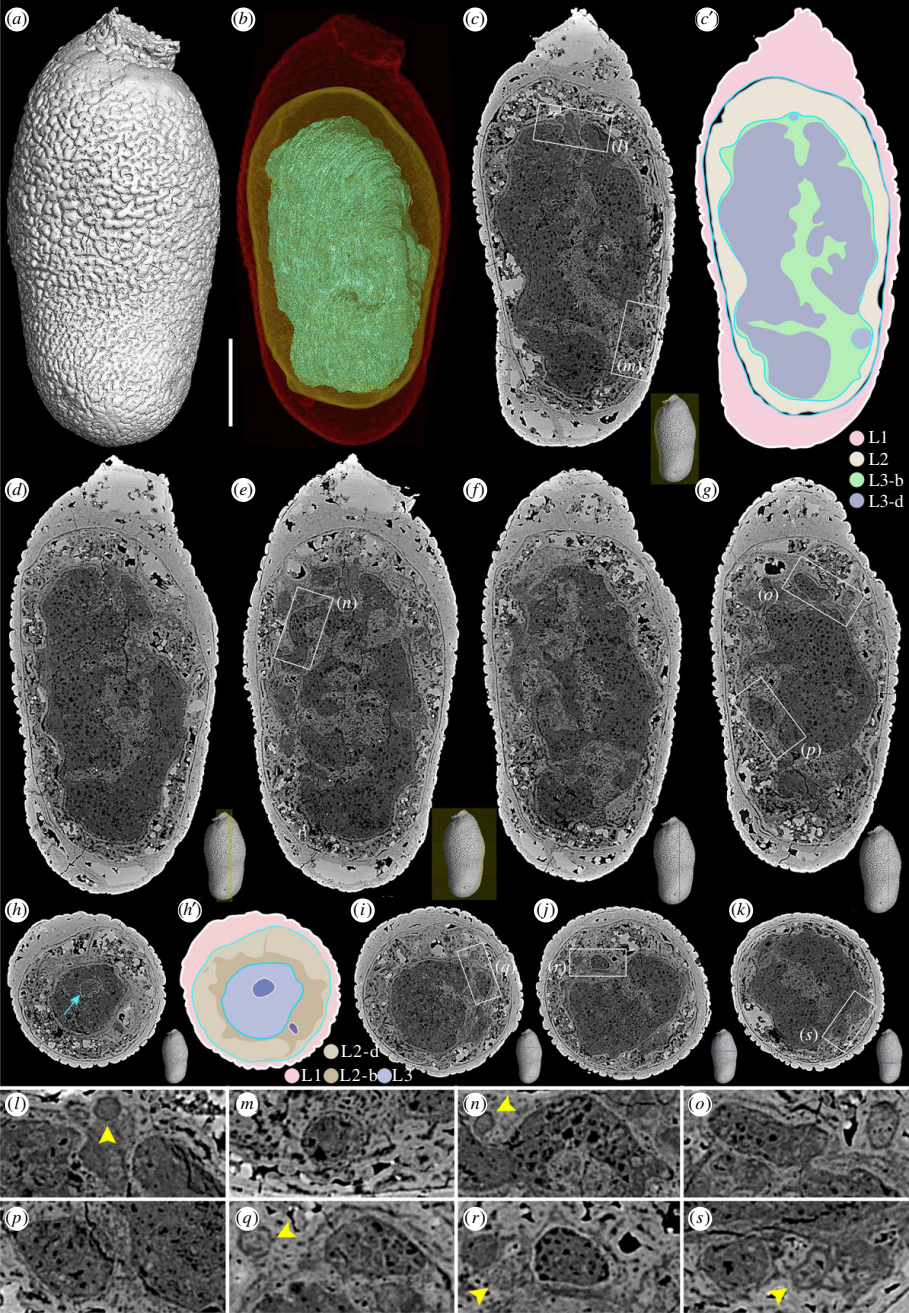
Elongate embryo-like fossils with three concentric layers (L1–L3) and cell differentiation. (*a*) Surface rendering. (*b*) Transparent model showing three layers: outer layer in red, middle layer in yellow and inner layer in cyan. (*c*–*g*) Longitudinal slices showing internal structures. (*h*–*k*) Cross-section slices showing internal structures. The outlines of the three layers in (*c*) and (*h*) are displayed in (*c*′) and (*h*′), respectively. (*l*–*s*) Close-up views of the framed areas in (*c*), (*e*), (*g*), (*i*), (*j*) and (*k*), showing details of multicellular structures in inner layer. The arrow in (*h*) indicates a membrane-bounded cell cluster, while the yellow arrowheads in (*l*), (*n*), (*q*), (*r*) and (*s*) indicate spherical structures. The scale bar represents 250 μm for (*a*) to (*g*), 290 μm for (*h*) to (*k*); 80 μm for (*l*) to (*o*), and 75 μm for (*p*) to (*s*).

Here we present a collection of EELFs bearing elongate olive- or peanut-like shapes (figures 4 and 5). Tomographic reconstructions suggest that some specimens were permineralized at early cleavage stages. As illustrated in figure 4, three specimens are of the 2-cell stage (figure 4*a*–*k*) while another one is of the 8-cell stage (figure 4*l*–*t*). All the specimens at different cleavage stages are similar to each other in size, around 1 mm long and 0.4–0.5 mm wide, suggesting that they were undergoing palintomic cell division. These elongate cleaving specimens have an ornamented envelope (figure 4*g*,*h*), similar to those in spherical cleaving EELFs reported previously (electronic supplementary material, figure S1 K–Q). Nevertheless, many cleaving EELFs had secondarily lost their ornamented envelopes (figure 4*a*–*f*,*l*,*m*). The two cells of the specimens in figure 4*a*–*k* detach from each other after cytokinesis. Such a feature resulted from degradation during post-mortem processes. This interpretation is supported by taphonomic experiments showing that embryonic cells of living sea urchins became rounded and disaggregated during initial degradation [43]. In the specimen displayed in figure 4*n*–*t*, the cleaving cells with tightly sutured polyhedral shapes maintain their original geometric relations.

Some exquisite intracellular structures have been preserved in these cleaving specimens. For example, the two cells of the specimen in figure 4*g*–*k* contain many small spherical structures (figure 4*j*,*k*,*w*, *x*; electronic supplementary material, movies S1 and S2), which are *ca* 10–20 µm in diameter and were interpreted as lipid drops or yolk granules [23]. The cells of the specimen displayed in figure 4*l*–*t* preserved nuclei, one for each (figure 4*s*,*t*,*y*,*z*; electronic supplementary material, movie S3).

In addition to these cleaving specimens, some other elongate EELFs exhibit three concentric layers (electronic supplementary material, figures S4 and S5). One typical specimen shown in figure 5*a* is 1.2 mm long and 0.4–0.5 mm wide with a sculptured surface. High-resolution tomographic reconstruction and scanning electron microscopic (SEM) observation suggest that the specimen is tri-layered with membrane-like boundaries between the three layers (figure 5*b*–*k*; electronic supplementary material, figure S4A,C,D). The outer layer (red in figure 5*b*; L1 in figure 5*c*′,*h*′) consists of three thin laminae (electronic supplementary material, figure S5A,B). The outer lamina has an ornamented surface and is uniform in thickness, while the middle lamina is not uniform in thickness. Underneath the middle lamina, there is the third lamina, a thin membrane-like structure (electronic supplementary material, figure S5B,E), which is *ca* 5 µm in thickness, separating the outer layer and the middle layer.

The inner layer looks like an olive-shaped core (cyan in figure 5*b*; L3 in figure 5*c*′,*h*′) with an undulating surface defined by a membrane-like structure (figure 5*c*). Intriguingly, this layer differentiated into dark (L3-d in figure 5*c′*) and bright (L3-b in figure 5*c*′) areas with distinct grey values. Energy-dispersive X-ray spectroscopy (EDS) elemental mapping indicates that the specimens are almost homogeneous in their chemical composition, with both the dark and bright areas being phosphatized (electronic supplementary material, figure S6). However, a slight difference in Ca depletion and C enrichment in the inner layer (electronic supplementary material, figure S6A2–A3) was observed, implying that possible organic remains (e.g. kerogen) were preserved in the inner layer. Though the signal of C could be altered by the surrounding epoxy, the Ca depletion supported this inference. Therefore, both the chemical composition and the packing density of the initial textures of the biological structures which have been permineralized by nanometre-sized, randomly oriented apatite crystals (electronic supplementary material, figure S5) that render the contrast seen in X-ray and electron images. In the light of high-resolution tomography, multicellular structures of the inner layer have been identified (figure 5*c*–*s*; electronic supplementary material, movie S4). However, the multicellular structures are not uniform since many ‘cell clusters’ have developed (figure 5*h*,*l*–*s*). These cell clusters were membrane-bounded and separated from the matrix. Additionally, a number of spherical vesicles with different sizes can be observed in this layer (figure 5*l*,*n*,*q*,*r*,*s*, arrowheads). These vesicles are unlikely to be cellular and are generally smaller than the cell clusters.

The middle layer (yellow in figure 5*b*; L2 in figure 5*c′*,*h*′) has decayed much relative to the other two layers. However, remains of the biological structures of this layer can still be observed (figure 5*d*–*h*; electronic supplementary material, movie S4). More specimens with three concentric layers are displayed in electronic supplementary material, figure S4, and some of them show well-preserved middle layers without typical void-filling mineralization but with multicellular structures (e.g. electronic supplementary material, figure S4F–J,K–P).

We propose that the elongate EELFs with palintomic cell cleavage in figure 4 are of early developmental stages, while the specimens possessing tri-layered architecture (electronic supplementary material, figures S4 and S5) have developed into late stages with cell differentiation. The elongate EELFs have been assigned into a life cycle of spherical *Megaspheara* (type 1 EELFs) as late developmental stages undergoing germination or propagule release [22]. However, our new evidence of parallel early cleaving stages shows that they are not late developmental stages of spherical *Megasphaera*.

## 3. Discussion

### (a) Cell division patterns, developmental sequences and subcellular structures reflect biology rather than geology

Nearly all of the phosphatized EELFs from the Weng'an Biota have undergone taphonomic and diagenetic processes [44]. Indeed, the shapes and sizes of cells could be altered to some extent during post-mortem and diagenetic processes, and EELFs with bad quality of preservation are quite common in the Weng'an Biota [14]. However, the logic of these arguments cannot be used to deny that biological features could be preserved in some specimens with high fidelity. For example, the regular cell arrangements and regular changes of cell sizes in the four types of EELFs characterized by distinctive CDPs, as well as the subcellular structures including nuclei (in type 1 and type 2 EELFs) and nutritional granules (in type 4 EELFs) (table 1), suggest that they did not suffer much from diagenetic alteration. The CDPs and development sequences do represent biological features rather than geological artefacts, because taphonomic and diagenetic processes can destroy rather than generate regular cell arrangements [43]. In particular, we believe that the post-mortem or diagenetic processes cannot generate complex structures such as the bilaterally symmetric arrangement of cells in type 3 EELFs (figure 2) and the dynamic development of type 4 EELFs (figure 3). Furthermore, some complex developmental patterns of EELFs with polar-lobe formation [8,15] and meroblastic cleavage [16] reported previously cannot be satisfactorily interpreted as diagenetic artefacts. Some cleaving EELFs with more than three cells could generate artefacts similar to polar-lobe formation by losing one or more cells during post-mortem processes, but in these cases, they lack a neck-like structure (polar-lobe neck) bridging the cytoplasmic polar lobe and the host cell [15]. Furthermore, post-mortem decay cannot produce the whole developmental sequence of polar-lobe formation observed in the EELFs [8,15]. Quantitative analysis showing linear relationship between the sizes of polar lobes and the cells at different developmental stages is also against the interpretation of diagenetic artefacts [8].

**Table 1.** Characters of diverse Weng'an Ediacaran embryo-like fossils (EELFs).

EELFs	shape	envelope structure	ornaments	subcellular structures	cleavage pattern	cell spatial geometry	abundance	reference
type 1 *Megasphaera*	spherical	single-layered	ornamented/smooth	nuclei and granules	equal and synchronous	spiral-like	abundant	this study; [4]
type 2 *Megasphaera*	spherical	single-layered	ornamented/smooth	nuclei and granules	equal and asynchronous	spiral-like	common	this study; [23,40]
type 3 *Megasphaera*	spherical	?	?	?	unequal and asynchronous	bilaterally symmetric	rare	this study
type 4 *Megasphaera*	spherical	bi-layered	ornamented	granules	unequal and asynchronous	polarity (body axis)	abundant	this study; [6]
type 5 *Megasphaera*	peanut-like	single-layered	ornamented	nuclei and granules	equal and synchronous	spiral-like	abundant	this study
type 6^a^ *Megasphaera*	spherical	single-layered	ornamented?	?	equal and synchronous	?	abundant	[24]
*Caveasphaera*	spherical	bi-layered	ornamented	?	?	?	abundant	[36]
*Helicoforamina*	spherical	bi-layered	ornamented, helical loop	nuclei	equal and synchronous	spiral-like	abundant	[35]
*Spiralicellula*	spherical	single-layered	ornamented	nuclei and granules	equal and synchronous	spiral-like	abundant	[37,40]

^a^Type 6 = *Megaclonophycus*-stage *Megasphaera* with matryoshkas [24].

In summary, we argue that all the CDPs mentioned above and the developmental sequences of type 1 and type 4 EELFs cannot be explained as diagenetic artefacts; more likely, they provide critical evidence of developmental biology. Up to now, the diverse CDPs of these EELFs have been largely overlooked; therefore, the developmental mechanisms and associated phylogenetic implications underlying the diverse CDPs have been grossly underestimated.

### (b) Associated developmental mechanisms and phylogenetic implications of cell division patterns

During the last two decades, continuing discoveries have shed new light on the biology of EELFs. For example, the exceptional preservation of cell nuclei and complex ornamented envelopes provide strong evidence of eukaryotic origin for the EELFs [12,13,40], and this viewpoint has been widely accepted [18,26]. Recent studies on the EELFs including *Caveasphaera* and *Helicoframina* rejected algal interpretations and attributed them to be holozoan with strong confidence [35,36]. Within the tree of Holozoas, three competing hypotheses on the nature of these EELFs have crystallized, i.e. non-metazoan holozoans [22], stem-group metazoans [18,23,24] or crown-group metazoans [8,16]. The well-preserved EELFs with diverse CDPs and developmental processes presented here and previously reveal that their development involved several specific biological features (tables 1 and 2), including *palintomic cleavage* (all the Weng'an EELFs), complex envelopes (all the Weng'an EELFs), *maternal nutrition* (e.g. type 1 [23], type 4, type 5 and *Caveasphaera* [36]), *asymmetric cell divisions* (e.g. type 3, type 4 and polar-lobe-forming EELFs [8,15]), *symmetry breaking* (e.g. type 4 and polar-lobe-forming EELFs [8,15]), *establishment of polarities or axes* (e.g. type 3, type 4 and meroblastic EELFs [16]), *spatial cell migration* (e.g. type 4 and *Caveasphaera* [36]) and *spatial cell differentiation and separation* (e.g. type 5 EELFs and EELFs with ‘matryoshkas’ [24]). This suite of features offers critical evidence to test the competing hypotheses for the EELFs.

**Table 2.** Developmental mechanisms of diverse Weng'an Ediacaran embryo-like fossils (EELFs).

EELFs	development feature	reference
type 1 *Megasphaera*	palintomic cell division, complex envelope, maternal nutrition	this study; [4]
type 2 *Megasphaera*	palintomic cell division, complex envelope, maternal nutrition	this study; [23,40]
type 3 *Megasphaera*	palintomic cell division, asymmetric cell division	this study
type 4 *Megasphaera*	palintomic cell division, complex envelope, maternal nutrition, asymmetric cell division, symmetry breaking, polarity (body axis), spatial cell migration	this study; [6]
type 5 *Megasphaera*	palintomic cell division, complex envelope, maternal nutrition, spatial cell differentiation and separation	this study
type 6^a^ *Megasphaera*	palintomic cell division, complex envelope, spatial cell differentiation and separation, programmed cell death	[24]
*Caveasphaera*	palintomic cell division, complex envelope, maternal nutrition, symmetry breaking, polarity (body axis), spatial cell migration	[36]
*Helicoforamina*	palintomic cell division, complex envelope, maternal nutrition	[35]
*Spiralicellula*	palintomic cell division, complex envelope, maternal nutrition	[37,40]

^a^Type 6 = *Megaclonophycus*-stage *Megasphaera* with matryoshkas [24].

Some extant non-metazoan holozoans (e.g. ichthyosporeans) use palintomic cell division to reproduce propagules [22,45]. In term of CDP, both equal and unequal cell division occur in different species [46], giving rise to specific cell arrangements that do look similar to type 1 or type 4 EELFs of certain stages. However, when the developmental processes (e.g. sequences of type 4) are taken into account, the similarity between them is much lower [45,46]. Moreover, there are no proper analogues for type 3 EELFs and meroblastic cleavage in terms of CDP. As reproductive cells, the propagules of ichthyosporeans aggregate within a cyst to form a temporal multicellular organism that normally lacks body polarity or axis. They do show temporally regulated cell type differentiation during life cycles [47–50], but spatial cell differentiation has not been reported in these metazoans' relatives [47,48,50]. On the contrary, the elongate EELFs (type 5) at later stages show distinct spatial cell differentiation and possible separation. Similar features interpreted as germ–soma separation have also been reported from some spherical EELFs with matryoshkas [24]. Even though we do not know whether the cell cluster separation in elongate EELFs represents germ–soma differentiation or not, these complex features characterized by spatial cell differentiation and separation never occur in any non-metazoan holozoans [24]. Hence, the diverse CDPs and associated developmental processes suggest that at least some types of these EELFs, if not all, are more complex than extant metazoans' unicellular relatives.

Some authors have hypothesized that the EELFs could be multicellular algae [25,51], even though no proper algal analogues have been found for these diverse EELFs [24,35,36]. Multicellular algae, including green, red and brown algae, have a great variety of morphology, developmental processes and life cycles; nevertheless, palintomic cleavage with a regular CDP only occurs in embryos of volvocine algae [25,26]. Though the early cleaving process of volvocine embryos could be broadly compared with type 1 EELFs to some extent [25], the former use cytoplasmic bridges to link the cells and this unique feature does not occur in any EELFs [24]. Furthermore, according to molecular clock estimates, volvocine algae probably arose during the Triassic, about 400 Myr later than the EELFs [52], and it is widely accepted that volvocine algae represent a recent independent origin of multicellularity [53]. In embryos of red and brown algae, palintomic cell division only occurs in several early rounds of cell division, and almost all red and brown algal embryos are naked or not free-living—none developed within a thick, ornamented envelope. Obviously, this is not the case for the EELFs. Different from the algal thalli from the Weng'an Biota, which are always naked [54], the type 5 EELFs at late stages with complex cell differentiation and cell cluster separation still lived within a very thick, multi-layered envelope, suggesting that they are unlikely to be multicellular algal thalli, because algal thalli need sunlight to survive, and obviously thick, multi-layered ornamented envelopes are not good for photosynthesis.

Within the extant animal kingdom, the diverse embryonic cleavage patterns with various development processes provide appropriate analogues for the EELFs. From the perspective of geometric relationships of cleaving cells, the CDPs of type 1 and type 2 EELFs follow the geometry of spiral cleavage of animal embryos. Additionally, a similar early cleavage pattern can be found in some living sponges, for example *Spongilla lacustris* (demosponge) [19] (figure 1*l*–*q*) and *Halisarca dujardini* (demosponge) [6,20]. A slight difference is that the embryonic cells of *S. lacustris* aggregate loosely without strong compaction. In type 1 EELFs, cells were closely packed during early stages (i.e. *Parapandorina*-stage of *Megasphaera*), resulting in polyhedral geometries (figure 1), and at the late stage (i.e. *Megaclonophycus*-stage *Megasphaera*), as illustrated in electronic supplementary material, figure S7, cell compaction only occurred in the surface cell layer, leaving the internal cells loosely aggregated. Therefore, the closely packed surface cells have faceted shapes without gaps in between (electronic supplementary material, figure S7A–C), while the internal cells are round with detectable intercellular space (electronic supplementary material, figure S7D). The morphological variation between the surface and internal cells has been interpreted as the onset of cell differentiation, and the compacted polygonal surface cells may be the precursors of epithelia [24].

The type 3 EELFs with a distinct cell arrangement characterized by bilaterial symmetry reflect precise control over the orientation of the cell division planes, and a bilateral cleavage pattern also occurs in embryos of extant animals such as tunicates [55]. The developmental sequence of the type 4 EELFs is comparable to embryonic developments of certain living animals; for example the embryos of *Asplanchna ebbesbornii* (rotifer) have a very similar developmental process with unequal and asynchronous cleavage (figure 3*t*1–*t*8) [42]. Furthermore, they also show striking similarities in envelopes and subcellular structures. The embryos of *A. ebbesbornii* have ornamented, bi-layered envelopes (electronic supplementary material, figure S2E–G) and numerous spherical yolk granules (figure 3*t*1) in the embryonic cells [42]. Comparable bi-layered, ornamented envelopes (electronic supplementary material, figure S2A–D) and subcellular granules (electronic supplementary material, figure S3) have been observed in the type 4 EELFs. In type 5 EELFs, membrane-bounded cell clusters developed within the inner layer, suggesting possible cell differentiation and separation. If the middle layer proves to be a biological structure rather than a diagenetic artefact, this organism may have developed two cellular layers.

**Figure 6.**
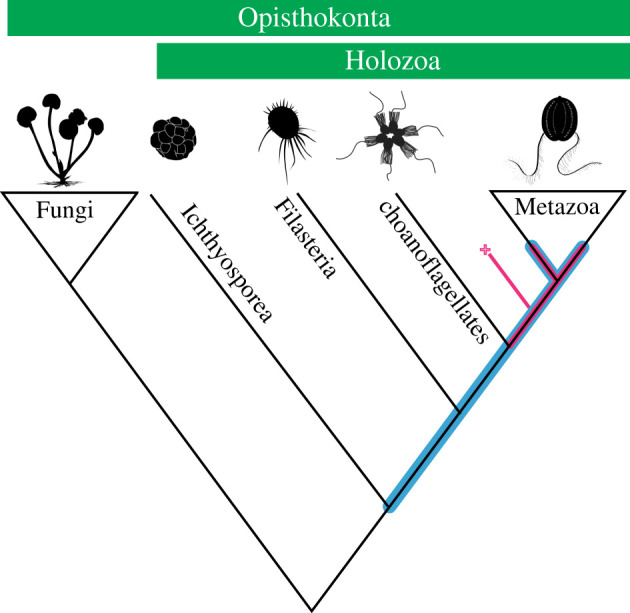
A simplified phylogenetic tree of the Holozoa, with Fungi as the outgroup. The Weng'an EELFs were pinned to the holozoan tree with a loosely constrained range of positions (indicated in blue) [35,36]. In this study, the potential placements for the Weng'an EELFs with complex developmental processes (e.g. the type 4 EELFs, type 5 EELFs, type 6 EELFs with matryoshkas, *Caveasphaera* and *Helicoforamina*) in the holozoan tree are indicated in red (total-group metazoans). The clade marked by a cross indicates extinct relatives of crown-group metazoans.

In summary, the diverse CDPs, developmental sequences and associated development features mentioned above suggest that some Weng'an EELFs, if not all, are more complex than the extant unicellular relatives of metazoans in terms of developmental biology. This conclusion has also been supported by detailed investigations on *Caveasphaera* [36] and *Helicoforamina* [35]. In this context, some Weng'an EELFs with more complex developmental processes and biological features (e.g. the type 4 EELFs, type 5 EELFs, type 6 EELFs with matryoshkas, *Caveasphaera* and *Helicoforamina*) are in favour of the total-group metazoan scenario (red line in figure 6), because the combination of these complex features (tables 1 and 2) can only be found in metazoans or stem-group metazoans [26,27,47,48]. Nevertheless, it is hard to further constrain the phylogenetic positions for some simpler Weng'an EELFs (for example the type 1 EELFs, type 2 EFLFs and *Sporosphaera* [56]) within the holozoan tree (blue line in figure 6), because we still cannot completely reject the possibility that these simple EELFs could be extinct metazoans' unicellular relatives (non-metazoan holozoans), given that they bear no unambiguous apomorphic characters of total-group metazoans based on current evidence.

Contrary to taking all the Weng'an EELFs to be one clade with the same affinity [18,22,23,25,26,51,57], we propose that these Weng'an EELFs probably represent various clades with different phylogenetic positions within the holozoan tree. Developmental biology of *Helicoforamina* revealed that the diversity of these Weng'an EELFs is much higher than previously thought [35]; however, how diverse they are remains unclear. We argue that the EELFs represented by different developmental mechanisms highlight the biological diversity of the EELFs from the Weng'an Biota. Given that the EELFs contain a variety of forms representing different clades within the holozoan tree, and some of them can even be pinned to the total-group metazoan tree, they provide us a unique window to test the ‘evo-devo’ hypotheses on the origin of metazoans and their embryology as well.

## 4. Material and methods

All the EELFs for this study were collected from the grey facies of the upper phospharite member of the Ediacaran Doushantuo Formation in Weng'an, Guizhou Province, southwest China [58]. The phosphatized dolomite from the grey facies was digested using approximately 7–10% acetic acid, and the fossils were manually sorted from the residues under a binary stereomicroscope. A set of high-spatial-resolution techniques, including propagation phase-contrast synchrotron radiation X-ray microtomography (PPC-SRXMT), high-resolution micro-CT (hr-μCT), SEM, EDS, focused ion beam SEM (FIB-SEM) and transmission electron microscopy (TEM), was used to obtain detailed physical and *in situ* chemical information from the EELFs.

### (a) SEM-EDXS, FIB-SEM and TEM

To select well-preserved specimens, all the fossils liberated from rock matrix were first investigated using a Leo 1530VP SEM instrument operating at 10 kV equipped with a field emission gun, located in the Nanjing Institute of Geology and Palaeontology, Chinese Academy of Sciences (NIGPAS). To perform chemical composition analysis and TEM observation, several fossil specimens were embedded in UV-cured resin and then cut after tomographic reconstruction. We observed the physical sections using a field emission FEI Nova NanoSEM 450 microscope (FEI, Hillsboro, OR, USA) operating at 15 kV at the Electron Microscopy Lab (EML), Institute of Geology and Geophysics, Chinese Academy of Sciences (IGG-CAS). The elemental mapping was performed using an EDS system (Oxford X-Max 80) attached to the FEI Nova NanoSEM 450 microscope. The ultrathin sections for TEM observations were prepared with a dual-beam FIB-SEM system on a Zeiss Auriga Crossbeam instrument at the EML of IGG-CAS. TEM analyses were carried out on a JEM2100 microscope (JEOL, Tokyo, Japan) operating at 200 kV, located in the EML of IGG-CAS.

### (b) PPC-SRXMT and hr-μCT

Selected specimens were imaged using scanning electron microscopy first, and the well-preserved ones were then scanned at Beamline ID19 of the European Synchrotron Radiation Facility (Grenoble, France) using PPC-SRXMT, or at the MicroCT Lab of NIGPAS using hr-μCT.

#### (i) PPC-SRXMT

We used an undulator source which can deliver a single harmonic X-ray with energy 17.68 keV. The relative monochromaticity of the beam is so good that it is not necessary to use a monochromator. Depending on the sizes of the fossil specimens, two CCD-based high-resolution detectors with isotropic voxel sizes of 0.56 and 0.70 µm were applied. During each scan, 1800 projections over 180° were collected. The exposure time for each projection was 0.2 s. In order to get a phase-contrast effect, 10 and 12 mm were adopted as the propagation distances (sample–detector distance). In addition to the simple edge detection mode, we applied a single distance phase retrieval process [16] for some of the fossils.

#### (ii) hr-μCT

We used a three-dimensional X-ray microscope (3D-XRM), Zeiss Xradia 520 versa, which can provide nondestructive reconstructions of microfossils at submicron resolution. Unlike conventional microCT, which relies on maximum geometric magnification and a flat-panel detector to achieve high resolution, 3D-XRM uses charge-coupled device (CCD)-based objectives to get higher spatial resolution. Depending on the sizes of the fossil specimens, two CCD-coupled 4× and 20× objectives were employed, providing isotropic voxel sizes from 0.55 to 0.80 µm. The operating voltage for the X-ray tube was set to be 40–60 kV. During each scan, 3200 projections over 360° were obtained, and a thin X-ray filter (LE1) was used to avoid artefacts of beam hardening. Owing to the low intensity of the X-rays, the exposure time for each projection was relatively long, from 5 to 8 s for different scans.

### (c) Volume data analyses

Volume data processing, including three-dimensional volume renderings, ‘ROI’ (region of interest) segmentation, and making animations, was performed using the software VGstudio Max (v. 2.2 and 3.0, Volume Graphics, Heidelberg, Germany).

## Data accessibility

All the fossil specimens were deposited at the Nanjing Institute of Geology and Palaeontology, Chinese Academy of Sciences (NIGPAS). The supporting tomographic data are available from the three-dimensional model database of NIGPAS (http://fossil-ontology.com, doi:10.12091/fossil-ontology.20211230). All data needed to evaluate the conclusions in the paper are present in the paper and/or the electronic supplementary material [59].
